# Effect of the telemedicine-supported multicomponent exercise therapy in patients with knee osteoarthritis: study protocol for a randomized controlled trial

**DOI:** 10.1186/s13063-023-07749-4

**Published:** 2023-11-14

**Authors:** Yuan Feng, Yan Wu, Huizhen Liu, Tianjie Bao, Chongyang Wang, Zezhang Wang, Jielei Huang, Yiwei Jiang, Chengqi He, Siyi Zhu

**Affiliations:** 1 Department of Rehabilitation Medicine, West China Hospital, Sichuan University, Chengdu, China; 2 Department of Rehabilitation Medicine, West China Clinical Medical College, Sichuan University, Chengdu, China; 3 Department of Postgraduate Students, West China School of Medicine/West China Hospital, Sichuan University, Chengdu, China; 4 College of Marxism, Sichuan University, Chengdu, China; 5 Centre for Biostatistics, Design, Measurement and Evaluation (CBDME), West China Hospital, Sichuan University, Chengdu, China; 6 Rehabilitation Key Laboratory of Sichuan Province, West China Hospital, Sichuan University, Chengdu, China; 7 Department of Computer Science and Technology, Tsinghua University, No. 30, Shuangqing Road, Beijing, Haidian District China

**Keywords:** Knee osteoarthritis, Telemedicine, Randomized controlled trials

## Abstract

**Introduction:**

The rising prevalence of knee osteoarthritis is placing a considerable strain on the global healthcare system. To address this issue, telemedicine-supported multicomponent exercise therapy has emerged as a promising approach. This therapy combines exercise, patient education, and health coaching to empower knee osteoarthritis patients to manage their condition from the comfort of their homes. Nevertheless, there are some existing limitations in the current research on this approach, including challenges related to patient compliance and the absence of objective evaluation methods.

**Methods and analysis:**

Patients diagnosed with knee osteoarthritis, who have not undergone knee surgery in the past year, will be recruited for a randomized controlled trial. The trial will include an intervention group and a control group. The intervention group will receive an mHealth app-based multicomponent exercise therapy, consisting of exercise therapy, patient education, and health coaching. Meanwhile, the control group will receive usual care, involving drug therapy and patient education. The primary outcome of the trial will be the measurement of pain intensity, assessed using a visual analog scale at baseline and at 4, 8, and 12 weeks of the post-intervention. To analyze the data, a two-factor, four-level repeated measures ANOVA will be used if the assumptions of homogeneity of variance and sphericity are met. If not, a mixed effects model will be employed.

**Discussion:**

The aim of the study is to evaluate the effectiveness of multicomponent exercise therapy aimed at enhancing pain self-management for knee osteoarthritis patients in the comfort of their own homes. The intervention incorporate wearable devices equipped with advanced deep learning systems to monitor patients' adherence to the prescribed at-home exercise regimen, as well as to track changes in outcomes before and after the exercise sessions. The findings from this trial have the potential to enhance both the accessibility and quality of care provided to knee osteoarthritis patients, offering valuable insights for future improvements in their treatment and management.

**Trial registration:**

Chinese Clinical Trials Registry, ChiCTR2300073688. Registered on 19 July 2023, https://www.chictr.org.cn/bin/project/edit?pid=199707. World Health Organization International Clinical Trials Registry Platform, https://trialsearch.who.int/Trial2.aspx?TrialID=ChiCTR2300073688.

**Supplementary Information:**

The online version contains supplementary material available at 10.1186/s13063-023-07749-4.

## Administrative information

**Table:** 

Title {1}	Effect of the telemedicine-supported multicomponent exercise therapy in patients with knee osteoarthritis: study protocol for a randomized controlled trial
Trial registration {2a and 2b}	Chinese Clinical Trials Registry, ChiCTR2300073688. Registered 19 July 2023, https://www.chictr.org.cn/bin/project/edit?pid=199707. World Health Organization International Clinical Trials Registry Platform, https://trialsearch.who.int/Trial2.aspx?TrialID=ChiCTR2300073688
Protocol version {3}	Version 12 of 14–10-2023
Funding {4}	This study is supported by the National Natural Science Foundation of China (81972146; 82002393; 82272599), National Innovation and Entrepreneurship Training Project for Undergraduate (S202310610541), Sichuan University Postgraduate Education Reform Project (GSSCU2021038; GSSCU2021130), and Sichuan Provincial Health Commission Universal Application Project (20PJ018). The funders play no role in the design, conduct, or reporting of this study.
Author details {5a}	Yuan Feng: ^1^Department of Rehabilitation Medicine, West China Hospital, Sichuan University, Chengdu, China ^2^Department of Rehabilitation Medicine, West China Clinical Medical College, Sichuan University, Chengdu, China Yan Wu: ^3^Department of Postgraduate Students,West China School of Medicine/West China Hospital, Sichuan University, Chengdu, China ^4^College of Marxism, Sichuan University, Chengdu, China Huizhen Liu: ^5^Centre for Biostatistics, Design, Measurement and Evaluation (CBDME), West China Hospital, Sichuan University, Chengdu, China Tianjie Bao: ^1^Department of Rehabilitation Medicine, West China Hospital, Sichuan University, Chengdu, China ^2^Department of Rehabilitation Medicine, West China Clinical Medical College, Sichuan University, Chengdu, China ^6^Rehabilitation Key Laboratory of Sichuan Province, West China Hospital, Sichuan University, Chengdu, China Chongyang Wang: ^7^Department of Computer Science and Technology, Tsinghua University, No. 30, Shuangqing Road, Haidian District, Beijing, China Zezhang Wang: ^1^Department of Rehabilitation Medicine, West China Hospital, Sichuan University, Chengdu, China ^2^Department of Rehabilitation Medicine, West China Clinical Medical College, Sichuan University, Chengdu, China Jielei Huang: ^1^Department of Rehabilitation Medicine, West China Hospital, Sichuan University, Chengdu, China ^2^Department of Rehabilitation Medicine, West China Clinical Medical College, Sichuan University, Chengdu, China Yiwei Jiang: ^1^Department of Rehabilitation Medicine, West China Hospital, Sichuan University, Chengdu, China ^2^Department of Rehabilitation Medicine, West China Clinical Medical College, Sichuan University, Chengdu, China Chengqi He: ^1^Department of Rehabilitation Medicine, West China Hospital, Sichuan University, Chengdu, China ^2^Department of Rehabilitation Medicine, West China Clinical Medical College, Sichuan University, Chengdu, China ^6^Rehabilitation Key Laboratory of Sichuan Province, West China Hospital, Sichuan University, Chengdu, China Siyi Zhu: ^1^Department of Rehabilitation Medicine, West China Hospital, Sichuan University, Chengdu, China ^2^Department of Rehabilitation Medicine, West China Clinical Medical College, Sichuan University, Chengdu, China ^6^Rehabilitation Key Laboratory of Sichuan Province, West China Hospital, Sichuan University, Chengdu, China
Name and contact information for the trial sponsor {5b}	Investigator-initiated clinical trial Chengqi He (Principal Investigator) hxkfhcq2015@126.com
Role of sponsor {5c}	This is an investigator-initiated clinical trial. Therefore, the funders play no role in the design of the study and collection, analysis, and interpretation of data and in writing the manuscript.

## Introduction {6a}

Knee Osteoarthritis (KOA) is a prevalent global disability disease and represents the most common form of arthritis. Individuals living with KOA often endure symptoms such as pain, swelling, and stiffness around the knee, primarily caused by the degradation of articular cartilage or injury to the subchondral bone [1]. The worldwide impact of KOA is substantial, affecting over 260 million people, with a notable increase of 9.3% between 1990 and 2017 [2]. It is estimated that radiographically confirmed symptomatic KOA accounts for 3.8% of all KOA cases, with the prevalence escalating with age, surpassing 10% among individuals aged 60 and above [3]. This upward trend in KOA prevalence is projected to persist due to aging populations and the growing rates of obesity, placing a significant burden on society. A key objective in the treatment of knee osteoarthritis (KOA) is the reduction of pain and improvement of functioning. To address these goals, treatment approaches should target factors contributing to pain and physical function. Guidelines recommend first-line treatments such as exercise, education, weight loss, health coaching, self-management, and psychosocial support [4–6]. Studies have demonstrated that multicomponent exercise therapy provides greater benefits to KOA patients compared to single physical therapy interventions [7, 8]. This comprehensive approach addresses the dysfunction and mental health of KOA patients from a socio-biologic-psychological perspective, resulting in improvements in peripheral muscle strength, joint motion, and central motor control. Additionally, health coaching plays a significant role in changing patients' pain perception and reducing fear of exercise. By offering an effective means for KOA patients to enhance their quality of life and reintegrate into society, this multi-modal therapy holds promise for improved outcomes [9, 10].

Despite the strong recommendation for multicomponent exercise therapy in the guidelines for knee osteoarthritis (KOA) management, its widespread implementation faces challenges due to limited consultation time and the labor-intensive nature of the approach. KOA is a chronic musculoskeletal pain syndrome that necessitates long-term treatment. However, practical limitations such as geographical barriers, transportation issues, and time constraints often result in patients having to self-manage their condition at home [11]. The advent of telemedicine has presented an opportunity to enhance the utilization of multicomponent exercise therapy for the home-based self-management of knee osteoarthritis (KOA) patients. Telemedicine, a component of telehealth, involves the use of software-based, evidence-informed approaches for disease treatment, management, or prevention [12]. By harnessing the power of the internet, mobile devices, smartphones, websites, and other technologies, telemedicine can be utilized either independently or in conjunction with other therapies such as medications or medical devices to optimize patient care and improve outcomes. Accumulated evidence suggests that telemedicine holds the potential to improve treatment efficiency, overcome geographical barriers, reduce costs, and mitigate exercise-related injuries when conducted in the home setting [13, 14]. Based on our analysis of 20 randomized controlled trials, we found that telehealth-supported exercise programs resulted in significant improvements in pain and disability among knee osteoarthritis (KOA) patients [15]. As a result, the demand for telemedicine advice and interventions has witnessed a substantial increase [16]. However, it is important to acknowledge that older KOA patients may encounter challenges in utilizing telemedicine, such as difficulties in app registration and website logins. Additionally, telemedicine relies on remote monitoring, which means that patients receive follow-up care at home. This can limit outcome assessments to subjective measures like questionnaires, potentially introducing bias into the results [17]. Furthermore, ensuring compliance with exercise prescriptions is essential for the success of multicomponent exercise therapy, but currently, there is a lack of standardized objective tools to assess compliance.

Telemedicine holds promise in enabling multicomponent exercise therapy for knee osteoarthritis (KOA) patients at home. However, it is crucial to consider the specific requirements of older patients and the limitations associated with objective outcome measurement. To strengthen telemedicine-supported exercise programs for KOA, it is important to develop tools that can objectively monitor exercise compliance. Considering this, we have designed a randomized controlled trial that integrates telemedicine and exercise therapy for KOA patients using a user-friendly mobile app and wearable devices to track home-based exercises. The mobile app features a simple interface that is easy to use for individuals from diverse backgrounds. The app will record exercise duration and login times, providing an objective measure of patients' exercise compliance. Furthermore, the wearable devices will monitor joint movement during exercise, ensuring that the prescribed exercises are performed correctly and enabling quantitative evaluation of compliance. This trial aims to compare the outcomes of telemedicine-supported multicomponent exercise therapy (the trial group or TG) with usual care (the control group or CG). The primary goal is to assess whether the trial group exhibits better outcomes in terms of reducing the need for clinical care, decreasing pain levels, and alleviating activity limitations in KOA patients after they have completed standard conservative treatment.

## Methods

### Primary objective {7}

The primary objective of this study is to assess the effect of telemedicine-supported multicomponent exercise therapy on pain management in patients with knee osteoarthritis in comparison to usual care therapy.

### Secondary objectives {7}

i) Evaluate the effects of telemedicine-supported multicomponent exercise therapy on physical function and quality of life in knee osteoarthritis patients compared to usual care.
ii) Assess the impact of multicomponent exercise therapy on self-cognition, mental health status, and the reduction of pain fear and pain catastrophic imagery in patients with knee osteoarthritis.
iii) Determine the level of adherence to multicomponent exercise therapy among KOA patients who self-manage their condition at home.

### Design {8}

This study is a randomized controlled trial with two parallel groups, conducted in an open-label, difference-test manner, following a 1:1 allocation ratio, within a single center over a span of 12 weeks. Patients afflicted with knee osteoarthritis, visiting the outpatient physiotherapy clinic, will be extended an invitation to partake in the study. Post-eligibility screening, participants will be randomly assigned to either the treatment or control group. The trial aims to enroll 86 subjects. The study protocol will be articulated in accordance with the Standard Protocol Items: Recommendations for Interventional Trials (SPIRIT) guidelines [18]. A completed SPIRIT checklist can be located in Additional File 1.

### Participant timeline {13}

**Fig. 1:**
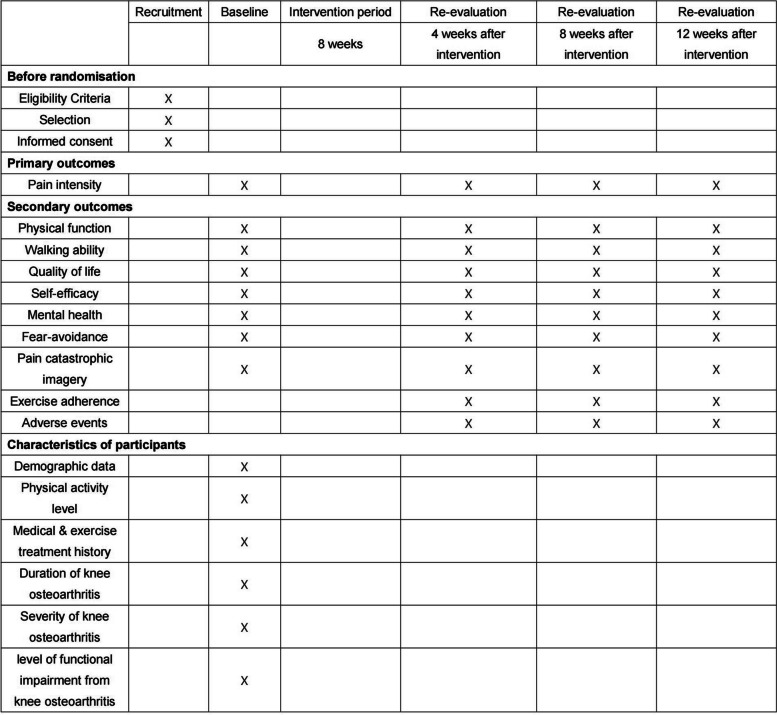
The schematic diagram of the participant timeline

This study will commence with the eligibility screening of prospective participants. Those meeting the eligibility criteria will be enrolled in the study and briefed on the procedures entailed. Participants deemed eligible and willing will be required to provide written informed consent (accessible in Additional File 2) prior to enrollment. We will assess their outcomes at the outset (W0), following 4 weeks of treatment (W5), 8 weeks of treatment (W9), and 12 weeks of treatment (W13). The schematic diagram illustrating the participant timeline is depicted in Fig. 1.

### Setting {9}

The study will begin recruiting participants in July 2023. Data collection and intervention administration are expected to conclude in July 2024. Participants will be recruited through Room 902, Special Needs Outpatient Department of West China Hospital, Sichuan University, Wuhou District, Chengdu City, Sichuan Province, China.

### Eligibility criteria {10]

If a patient expresses interest in participating, researchers will evaluate their eligibility based on the study's inclusion and exclusion criteria (Table 1).

**Table 1:** Inclusion and exclusion criteria

Inclusion criteria	Exclusion criteria
18 years old or older	Knee arthroscopy or open surgery in the past 12 months, elective knee replacement
Clinical signs and symptoms corresponding to KOA, according to the 2021 COA guidelines	With other joint problems (such as severe osteoporosis, rheumatoid arthritis, fracture, gout, joint tuberculosis, or joint tumor)
Kellgren-Lawrence Grade I–III of KOA	Malignant illness or other major conditions (e.g., unstable cardiovascular disorders or lung disease, dementia) that restrict the ability to adhere to the recommended treatment
Visual analog scale (VAS) score ≥ 4 points [19]	Cognitive impairment resulting in the inability to understand the physical therapist’s instructions and the content in the app
Access to smartphone or tablet	Pregnant women
Participate in the experiment voluntarily and sign the informed consent	The supervision and care of other family members cannot be guaranteed for each exercise training
Accept randomization assignment	Neurological disorders
Candidates who meet all the above criteria will be included	Candidates meeting any of the above criteria will be excluded

### Recruitment {15}

Participants for the trial will be recruited from Room 902, Special Needs Outpatient Department at West China Hospital, Sichuan University. Individuals meeting the eligibility criteria will be briefed on the trial procedure, following which, upon signing the informed consent form, they will be enrolled in the study. Those not meeting the eligibility criteria will be directed to the standard treatment protocol as per their preference. To reach the desired sample size, we will administer unified training and supervision to researchers to enhance the success rate of securing informed consent during interviews. Concurrently, we will employ other effective recruitment strategies, such as referrals from medical examination centers or relevant departments, and poster campaigns, to robustly ensure a swift recruitment pace of participants.

### Consent {26a}

A physical therapist will assess individuals who meet the aforementioned prerequisites to determine their eligibility for participation in the study. Initial study information outlining the trial’s background, appropriate population, interventions, risks and benefits of participating, along with rights and responsibilities, will be shared with potential participants by both physicians and researchers. Upon agreement to participate, individuals will receive detailed written information elucidating the study’s objectives and methodologies, and they will be required to sign three informed consent forms. One copy will be retained by the participants, another will be electronically scanned by the physiotherapist during the initial visit, while the third copy will be securely stored by the Ethics Committee of Sichuan University. The informed consent will be signed and obtained on the day of the first visit at Room 902, Special Needs Clinic, West China Hospital, Sichuan University.

### Additional consent provisions for collection and use of participant data and biological specimens {26b}

There are no additional biological samples to be collected.

### Randomization and blinding

#### Sequence generation {16a}

Block randomization with a computer-generated random sequence will be utilized to conduct randomization as participants are recruited. A designated data manager, who is not involved in recruitment, intervention, or evaluation processes, will be assigned to generate random sequences and store them securely in IBM SPSS, version 26.0.1.

Furthermore, the randomization list will be securely stored both digitally within a password-protected database, and physically in a secure, locked location within our facility. Only authorized personnel, such as the designated data manager and the principal investigator, will have access to the randomization list, ensuring its integrity throughout the course of the study. This setup not only safeguards the list but also allows for a structured protocol that remains unaffected by individual personnel changes.

#### Concealment mechanism {16b}

An independent researcher, not involved in the experiment, will follow the computer-generated random sequence results to insert the assignment codes into sequentially numbered, sealed, opaque envelopes, thereby obfuscating group allocation. As per the experimental design, once opened according to the stipulated procedures, participants will be assigned to either the “control group” or “trial group” based on the corresponding “0” or “1” on the random number card.

#### Implementation {16c}

During the recruitment phase, participants will be evaluated by a physical therapist who is not involved in the randomization process. Following this evaluation, sealed and opaque envelopes will be opened on-site to ascertain the assignment group, with participants thereafter being allocated to the respective groups in a 1:1 ratio. To ensure a balanced distribution among the groups, permuted blocks of size 4 will be employed randomly.

### Blinding

#### Who will be blinded {17a}

Blinding of volunteers and doctors to group assignments is unfeasible. The decision not to blind the assessors is made given that the subjects are not blinded and the outcomes are self-reported. Although participants, doctors, and assessors are not blinded, alternative methods are employed to minimize bias. Specifically, participants and assessors are not informed of the trial hypothesis to mitigate the risk of bias stemming from unblinding.

### Interventions

Qualified physiotherapists will provide treatment to participants in both groups but will not be involved in assessing and evaluating the outcome measures. The physiotherapy interventions for both groups will span 12 weeks, adhering to the recommendations from the 2019 guidelines established by the American Rheumatoid Arthritis Foundation [20]. Participants will be motivated to fully partake in the intervention and assessment processes throughout the trial. The physiotherapist administering the treatment will document details regarding participation rates, medication changes, and any adverse events during and after the treatment in the daily case report form.

### The explanation for the choice of comparators {6b}

Patients in the control group will receive usual care rehabilitation treatment, with app-based patient education (10 min per day, 1 day per week, for 12 weeks) and topical use of NSAIDs, diclofenac diethylamine latex (4 times a day, 7 days per week, for 2 weeks).

### Intervention description {11a}

#### TG therapy

The intervention measures for the trial group include app-based exercise therapy, patient education, and WeChat video-based health coaching.

#### App-based exercise therapy

**Fig. 2:**
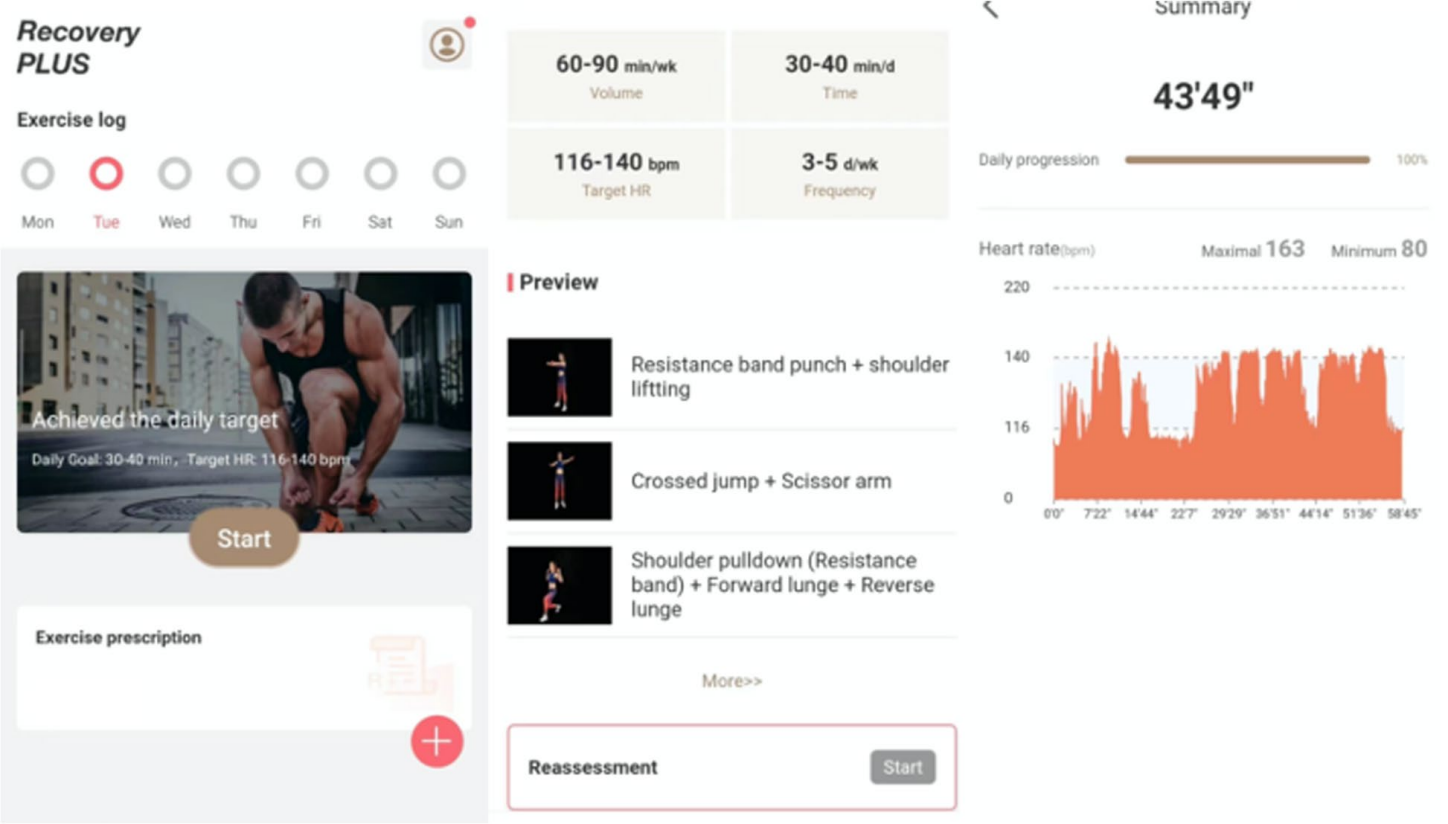
Examples of app content

The individuals will be given instructions by the researchers on how to download, sign up for, and utilize the app throughout the first diagnosis and therapy. In order to associate and bond with the doctor or therapist, patients must affirm that they have read the informed permission form, complete the personal information and questionnaire honestly, and then input the activation code. The patient can perform the training under the direction of the action video when the doctor approves the workout prescription. Based on the patient's age, gender, body mass index (BMI), lesion site, and degree, a personalized treatment strategy is adopted, with a focus primarily on quadriceps muscle training and neuromuscular training modules. According to the patient's preferences and practicality, the doctor or physical therapist chooses the most appropriate exercise for the patient to prescribe. The action training video, the frequency, the intensity, the length, and the overall quantity of exercise are all included in the prescription's fundamental material. Pictures, audio prompts, and wearable device monitoring are all integrated in the video instructions for the training exercises. The wearable device is equipped with Inertial Measurement Units (IMUs), which capture the acceleration, angular velocity, and orientation of the body part to which they are attached. This device is non-invasive and does not necessitate direct attachment to the skin. We are committed to enhancing the convenience and comfort for users when employing this device in their daily routines and will seek official medical device approval prior to public release. By capturing motion features, wearable device monitoring can record a patient's individual joint and determine whether the patient has completed the task. In addition, a deep learning system based on a large language model can describe the actions the patient is doing through changes in the node position, such as “this patient is extending his lower limbs in a standing position.” The number of movements, estimated length, movement preview, and written version of the movement instructions may all be seen prior to the activity beginning (shown in Fig. 2). You can start working out after successfully connecting to the wearable device after wearing it properly. The system will alert the patients that the standard has been attained when the exercise reaches the upper or lower limit of the intended duration, at which point they can decide whether to continue exercising or stop. The patients will be urged to meet the standard after the exercise, and they will be asked to rate their level of felt exertion. Based on patient input, the doctor will modify the recommended workout regimen. Through the alarm in the app, patients could set exercise time according to their own schedule for three times each week for 12 weeks, for a total of 36 training sessions. The software allows therapists to create staged goals in advance and remind patients on a regular basis whether they have achieved their goals or not.

The training moves are as follows:

Quadriceps exercises are designed to improve knee stability by increasing endurance and strength in major muscle groups. The therapist will make an exercise plan for the patient in advance according to the baseline measurement results, and import the training actions into the movement library of the app. According to the literature [21], the movements of training include (i) knee extension in a fixed range, (ii) sit and extend the knee, (iii) maintain the knee extension at 30° in the sitting position, (iv) straight leg elevation training, and (v) sitting elastic belt training.

Neuromuscular training aims to improve sensorimotor control to promote functional stability of the knee. According to the previous literature, the actions are as follows [22]: (i) core stability/postural function — static hip bridge with the help of a yoga ball; (ii) postural positioning — gliding forward and backward with the help of a horizontal bar or handrail standing position; (iii) lower limb muscle strength — knee flexion and extension muscle training in the sitting position with the help of an elastic band; (iv) functional exercise — stand and sit up training and up and down steps exercise.

Two to three sets of 10–15 repetitions will be performed for each exercise, with a 10–15-s rest before continuing to the next set of exercises. Subjects will be required to follow the app to complete the training sessions, 30 to 40 min each time, 3 times a week for 12 weeks, with a total of 36 training sessions.

### App-based patient education

During the baseline assessment, physical therapists will present patients with animated educational videos concerning knee osteoarthritis. Following this, patients will be directed to read through 12 illustrated educational articles on KOA on a weekly basis via an app [23]. Each article is estimated to require between 10 to 20 min to read through. It covers terms like definition, etiology, pathogenesis, diagnosis, treatment, day-to-day management, how to stop knee osteoarthritis from progressing and recurring, food management, weight management, and other topics. The questionnaire connected to knowledge of knee osteoarthritis will be provided through the app once every Friday in order to determine whether the patients actually read the information about the condition that was promoted by the app. The results of the questionnaire will be used to evaluate the patient’s learning situation.

### WeChat video-based health coaching

Once a week, a group WeChat video will be held (each video lasted 40 min), resulting in 12 out of 36 sessions being observed. At the same time, the physical therapist and the patient could speak about the development of the condition, diet, weight, and other matters, and the patients could communicate with each other. Health coaching based on group video can enable patients to provide peer aid and psychological support, improve therapist-patient adhesion, and increase trust between them. A weekly meeting can also be utilized to ensure that the patient’s motions are accurate and do not result in new injuries. If the patient’s motions are accurate and the progress markers are met, the therapist can decide to advance the patient's treatment plan during the meeting.

### CG Therapy

The intervention in the control group includes app-based patient education and topical use of NSAIDs, diclofenac diethylamine latex. Patients in the control group will be informed that they can receive TG therapy after the 12-week trial is over.

### App-based patient education

The treatment content is the same as that of the trial group. The app could set open permissions for patients, and the patients in the control group could only receive patient education knowledge once a week for 12 consecutive weeks, a total of 12 times. The exercise training plate is not open to it.

#### Drug therapy

Diclofenac diethylamine latex (GSK Consumer Healthcare S.A. Production, 20 g/piece, batch number DV6D) for external use, apply four times a day, respectively along the front of the affected area, inside and outside the order, gently rub to promote the absorption of the drug. The range of administration should be greater than 10 mm at the distal end of the knee joint. Patients should be instructed to avoid contact with clothing within 10 min of medication and avoid bathing or exercising within 1 h after medication and continue medication for 2 weeks [24].

### Criteria for discontinuing or modifying allocated interventions {11b}

Should a participant opt to withdraw from the trial prematurely, the physiotherapist will promptly reach out to ascertain the reason for their withdrawal. Participants who exit the trial, become unreachable for over 2 weeks, and fail to complete the outcome evaluation will be categorized as dropouts [25].

i) Patients desiring to cease participation;
ii) Patients unable to undergo the baseline assessment;
iii) Patients failing to complete the app-based exercise therapy sessions;
iv) Patients experiencing exacerbated symptoms.

### Strategies to improve adherence to interventions {11c}

To improve the patient’s adherence to the intervention protocols, we will use wearable device, through the change in joint angle, wearable device monitoring may record the patient's individual joints and determine if the patient has finished the task. Furthermore, once a week, a group WeChat video will be held. Health coaching based on group video can enable patients to provide peer aid and psychological support and improve therapist-patient adhesion.

### Relevant concomitant care permitted or prohibited during the trial {11d}

All participants will be provided with the same version of the app, which will remain unchanged throughout the trial. During the treatment phase, should a participant's condition deteriorate, they are permitted to utilize any relevant treatment to manage the disease, such as drug therapy or physical factor therapy, among others. However, engaging in additional exercise therapy is prohibited. Participants are required to report any such additional treatments accurately to the investigator, who will then document these in the case report forms.

### Outcome measures {12}

After obtaining informed consent, a baseline assessment will be conducted. The details gathered include participants’age (acquired using the birthdate shown on their ID cards), gender, height, weight, BMI, education level, occupation, income source, hobbies, interests, marital status, smoking and alcohol use, medical history, knee pain duration, pain severity (current, worst, mildest), triggers, alleviating factors, and pain-avoiding behaviors. The primary and secondary outcomes will be evaluated before commencing the trial (baseline) and at 4-, 8- and 12-week post-intervention (shown in Table 2). We focus on changes in outcomes at 12 weeks after treatment.

**Table 2:** Overview of the measurements and timing of measurements

Domain	Reporting methods	Questionnaire line	Base	4 weeks	8 weeks	12 weeks
Demographic characteristics	Self-reported	Purpose built	√	-	-	-
Pain intensity	Self-reported	VAS	√	√	√	√
Physical function	Self-reported	WOMAC	√	√	√	√
	Walking ability	TUG	√	√	√	√
Quality of life	Self-reported	SF-36	√	√	√	√
Self-efficacy	Self-reported	PSEQ	√	√	√	√
Mental health status	Self-reported	DASS21	√	√	√	√
Fear-avoidance in exercise	Self-reported	TSK	√	√	√	√
Pain catastrophic imagery	Self-reported	PCS	√	√	√	√
Exercise adherence	Self-reported	EARS	-	√	√	√
	Events	Movement completion rate	-	√	√	√
Adeverse events	Self-reported	-	-	√	√	√

#### Primary outcome measure

##### Pain intensity

A visual analog scale (VAS) is used to evaluate knee pain. Patients will be asked to rate their current pain level on a line of 100 mm in length, with 0 representing no pain and 100 representing extreme pain, and patients mark this point on the current pain line. The VAS score is then determined by measuring in millimeters from the left end of the line to the point marked by the patient. Patients can experience pain ranging from no pain to extreme pain. Scores range from 0 to 100, with higher scores indicating more severe pain [26].

#### Secondary outcome measures

##### Physical function assessment

The Osteoarthritis is assessed using the Western Ontario and McMaster Universities Osteoarthritis Index (WOMAC), a total of 24 questions, by patient self-evaluation, Scores range from 0 (no symptoms) to 4 (very severe) on a scale from 0 to 96, with higher scores indicating more severe pain and dysfunction [27].

##### Walking ability test

The time up go test (TUG) is a quick assessment of functional walking ability [28]. At the time of assessment, the patient will be seated in a backrest chair with armrests (seat height approximately 45 cm, armrest height approximately 20 cm), with the body resting on the back of the chair and hands resting on the armrests. If using a walker such as a cane, place your hand on the walker. Paste colored strips or draw a clearly visible thick line or place an obvious mark on the ground 3 m away from the seat. After the tester gives the “go” command, the patient stands up from the back of the chair, stands on his or her footing, walks forward 3 m with the usual walking gait, passes the thick line or mark, turns around, walks back to the chair, and then turns and sits down, leaning against the back of the chair. During testing, no help can be provided. The tester records the time in seconds for the patient to stand up from the starting position (reclining) and sit down again and lean back. The patient may be asked to practice several times to understand the content of the test before the formal test. In addition, the patient’s risk of falling during the test period will be determined. The time criteria are < 10 s, free movement; < 20 s, most can move independently; 20–29 s, unstable movement; and > 30 s, dyskinesia. The risk criteria are as follows: 1 for normal, 2 for very slight abnormalities, 3 for mild abnormalities, 4 for moderate abnormalities, and 5 for severe abnormalities [29].

##### Quality of life

The MOS item short-form health survey (SF-36) is used to assess the quality of life, which includes 8 dimensions: body function, physiological function, body pain, general health status, the vitality of life, social function, emotional function, and mental health, a total of 36 questions, according to the standardized weight of the corresponding score, the score of each question is added to get the test rough score, through the score operation conversion formula specified in the scale to get the final score, the total range of 0–100 points. The higher the final score, the higher the quality of life [30].

##### Patients' self-efficacy for pain

The Pain Self-Efficacy Questionnaire(PSEQ) is used to assess the confidence of patients with persistent pain to perform activities in pain [31]. There are 10 items in the questionnaire, and the evaluation standard for each item ranges from 0 to 7 points. 0 points represent no confidence at all, 7 points represent complete confidence, and higher scores indicate that patients have higher self-efficacy beliefs about returning to life. The reliability and validity of the questionnaire have been verified [32].

##### Mental health status

Mental health status is measured by Depression-Anxiety-Stress Scale (DASS21). DASS21 is a mental health assessment scale based on the three-factor model of depression, anxiety, and stress. The scale takes the degree of various negative emotional states induced by the subject as the evaluation index and uses a 4-point scoring method. Non-conforming to the standard is “0”; “1” means sometimes satisfied, “2” means often satisfied, “3” means always satisfied, and higher scores indicate higher depression, anxiety, and stress indices [33].

##### Fear-avoidance in exercise

Tampa Scale of Kinesiophobia (TSK) is a 17-item scale developed to assess fear of movement/re-injury. The scale includes parameters of injury/re-injury and fear-avoidance in work-related activities [34].

##### Pain catastrophic imagery

The Pain Catastrophizing Scale (PCS) is a widely adopted self-report tool for assessing exaggerated negative conceptualization of persistent, anticipated, or imaged pain. The PCS is a 13-item questionnaire consisting of three subscales (helplessness, magnification, and rumination), with each item on the scale being scored on a 5-point Likert scale, with a score of 0 representing “never” and 4 for “always,” with higher scores indicating higher levels of pain catastrophizing [35].

##### Exercise adherence

The Exercise Adherence Rating Scale (EARS) is a 16-item scale developed to evaluate the adherence of individuals to the exercises recommended for individuals with chronic pain diseases, and the reasons for their compliance or non-compliance. It consists of 3 subscales. Subscale A does not participate in scoring. Subscale B (evaluates self-reported adherence to exercises) consists of 6 questions and subscale C (evaluates the reasons for compliance or non-compliance with the recommended exercise) consists of 10 questions, and these subscales are scored [36]. In addition, we will calculate the completion rate of exercise prescriptions for patients with KOA by the number of completed movements captured by the wearable devices.

### Adverse events {22}

Any issue in the study knee or elsewhere in the body that the participant believes to be related to their participation in the trial is recognized to be an adverse event, as long as at least one of the following conditions is present: (i) that led to the participant seeking medical attention for 2 days or longer; and (ii) that exacerbated pain and/or disability [37]. Based on prior experiments, the incidence of serious adverse events such as death or sudden severe illnesses among patients undergoing exercise therapy is extremely low. In the case of adverse events necessitating emergency assistance (e.g., sudden cardiovascular issues, falls, and dizziness), the physiotherapists, doctors, and the rescue team at West China Hospital will provide prompt emergency aid. Should an emergency arise at home, our research team will have previously furnished the patient with contact information for, or access to, medical facilities in their community. These institutions will partake in providing the necessary care. Post-treatment, the clinical coordinator will document the timing and methods of intervention for the adverse events. In non-emergency scenarios, patients are to report adverse events to the clinical coordinator, who will in turn inform the physiotherapists and rehabilitation doctors within the research team. Following a consensus discussion, a viable resolution will be devised for the patient, and the clinical coordinator will relay this solution to the affected individual. The time span from report to feedback will not exceed 8 h. A consolidated report of adverse events will be featured in the final study report, detailing the type and frequency of such events.

### Sample size calculation {14}

The sample size was calculated by G*power 3.1.9 according to the following conditions. According to Cohen’s criterion for effect size [38], 0.2, 0.5, and 0.8 are the boundary values for small, medium, and large effect sizes, respectively. According to Mecklenburg test results [39], 0.3 was selected to verify the small effect size, α was 0.05, the test power was 0.8, and the intraclass correlation coefficient was 0.6. The experiment will be performed with a two-way repeated measures analysis of variance, and the two groups will be measured four times. The final calculated sample size was 64. Considering a dropout rate of 25%, the actual required sample size should be 86.

### Data collection and management

#### Plans for assessment and collection of outcomes {18a}

All examination forms will be printed and filled out by the assessor during each examination, with uniform training provided to the assessor to ensure greater consistency. The questionnaires will also be printed and completed by the patients. Data from the wearable device will be extracted from the app's backend by a statistician who is not involved in the trial intervention. Two data administrators will enter the data into anonymized tables that set up logical errors, such as mandatory fields and limits responses that violate common sense, and the data in the tables will subsequently be utilized for statistical analysis.

#### Plans to promote participant retention and complete follow-up {18b}

Patients will be given thorough information about the trial, including its design and needs, during the recruitment process. They will also be reminded of how crucial it is to finish the trial. Patients are free to stop participating in the study at any time and are not required to provide a reason. A quality form will be utilized to gauge the patient’s development, and patients will be reminded to assist with the researcher’s scale assessment at several points during the survey. Participants who have stopped or departed from intervention regimens will have their outcome information collected.

#### Data management {19}

All experimental procedures and data will be documented in a case report form by the outcome assessors. Each participant will be assigned a unique identification code to safeguard patient privacy. Access to the case report forms will be restricted solely to the outcome assessors and the corresponding author. All data entries will undergo a double-checking process by two independent assessors. Once entered and verified, the data within the case report forms will be rendered unmodifiable.

### Statistical analysis

#### Statistical methods for primary and secondary outcomes {20a}

The criteria of the Consolidated Standards of Reporting Trials (CONSORT) shall be followed in the calculation and reporting of summary data [40]. We will evaluate the descriptive features and baseline outcome metrics and baseline comparability between groups. During trial and follow-up, a variety of reasons can lead to patient drop-out and loss, so intention-to-treat analysis (ITT) and per-protocol analysis (PP) are required for data. ITT analysis refers to the analysis of the data obtained after the elimination of all randomly assigned subjects in a minimum and reasonable method, while PP analysis refers to the analysis of only the data of the observation objects who have completed the whole experiment after randomization [41]. However, ITT analysis prevents bias due to the subjects’ violation of protocol, loss of follow-up, and generally makes differences between the two groups less detectable and conservative; PP analysis, on the other hand, usually exaggerates the differences between the two groups. In order to avoid overstating the efficacy, ITT analysis will be used as the main analysis set in this study.

We will use IBM SPSS, version 26.0.1 for all statistical analyses, with 2-tailed tests as appropriate, and 2-sided *P* < 0.05 considered to be statistically significant. Baseline data on categorical variables (e.g., sex, occupation, education level) will be used to compare statistical differences between the trial and control groups using chi-square tests. For continuous variables (e.g., age, BMI, pain intensity, duration of knee pain), a normality test will be first conducted. If all groups meet normality, mean ± standard deviation (SD) will be used for statistical description, and two independent sample *T* tests or Mann–Whitney *U* tests will be used for comparison between groups. Otherwise, the median (interquartile spacing) will be used for statistical description, and a nonparametric test will be used for comparison between groups. If the results of the trial meet the conditions of homogeneity of variance and spherical symmetry, the two-factor four-level repeated measurement analysis of variance (group*time) is used; otherwise, the mixed effects model is used. According to the recommendations of the International Conference on Osteoarthropathy [42], the minimum clinically important difference (MCID) could be achieved when the pain intensity (primary outcome index: VAS) between the two groups is changed by 18 mm based on 100 mm. A chi-square test will be used to infer the proportion of the number of patients meeting the MCID between the two groups. The measurement results of all outcome indicators will calculate the effect size, and the size of the effect size between the two groups will be compared according to Cohen’s criteria [38] to draw the appropriate conclusions.

#### Interim analyses {21b}

There are no interim analyses planned.

#### Methods for additional analyses (e.g., subgroup analyses) {20b}

There are no subgroup analyses planned.

#### Methods in analysis to handle protocol non-adherence and any statistical methods to handle missing data {20c}

We will evaluate the loss of primary outcome data through intention-to-treat analysis. Missing data will be reduced to a minimum by using the multiple imputation.

#### Data monitoring {21a}

A Data & Safety Monitoring Committee is not necessary in this research since the interventions are low-risk and significant adverse events (incapacitating, life-threatening, hospitalization, or death) are incredibly improbable.

#### Composition of the coordinating center and trial steering committee {5d}

A group of individuals oversees this trial, including a principal investigator who is in charge of both the prosecution and medical treatment of the participants, two data managers who ensure data capture and quality, a study coordinator who manages registration and study visits, and a study physician who selects potential participants and oversees proper follow-up. Every Monday, the team meets; however, neither the trial steering committee nor a stakeholder/public involvement group are present.

#### Auditing {23}

Twice a month, independent supervisors make on-site visits to examine the existence and accuracy of the investigation files. Additionally, the monitoring team will check the following statistics on 20% of the patients who are chosen at random: Source data, inclusion and exclusion criteria, missing scales, reporting, and informed consent.

#### Ethics {24}

The study will be conducted in accordance with the provisions of the Declaration of Helsinki and has been approved by the Biomedical Ethics Committee of West China Hospital, Sichuan University, and is registered with the Chinese Clinical Trial Center.

#### Provisions for post-trial care {30}

Should any risks arise during the treatment phase, any adverse events attributed to the study will be managed by the project team at no cost to the participant. In the case of serious adverse events deemed to be associated with the clinical trial, the project team will provide the necessary treatment free of charge. For any financial losses incurred, the research group will offer corresponding monetary compensation, including a 300 yuan subsidy for transportation and accommodation, provided that the participant can furnish legally valid proof of loss.

#### Dissemination plans {31a}

The results will be reported in peer-reviewed journals, national and international conferences, and various media to share the findings with patients, clinicians, and the public.

#### Protocol amendments {25}

Significant alterations to the protocol will be submitted to the Biomedical Ethics Committee of West China Hospital, Sichuan University for approval. Patients engaged in the study are required to sign informed consent prior to their participation. In the event of any updates to the study protocol, patients will be requested to sign the updated informed consent form. Following this, notifications regarding the updated protocol will be disseminated to the researchers, General Practitioners, and the patients via email.

#### Confidentiality {27}

Research data will be marked with a special participant identification code throughout the trial. The study team will be the only ones with access to the key to this identification code list. The lead investigator will adhere to research protocols to record and secure the key after the study is finished. No information identifying a patient will be disclosed in publications.

## Discussion

Despite the proven benefits of multicomponent exercise therapy for knee osteoarthritis (KOA) in high-quality randomized controlled trials [43, 44], its implementation as a core treatment approach remains underutilized. Challenges such as limited outpatient treatment time and inadequate medical resources hinder the promotion of this model through traditional in-person doctor-patient communication in clinical practice. In response, the development of telemedicine-guided (TG) therapy offers an innovative solution for managing KOA patients. Previous studies have demonstrated that telemedicine interventions for KOA can be as effective as standard care [45, 46]. By leveraging telemedicine, there is an opportunity to establish a sustainable healthcare system that addresses the challenges associated with delivering optimal care for KOA patients.

The primary objective of this trial is to assess the reliability and sustainability of telemedicine-supported multicomponent exercise therapy in the management of knee osteoarthritis (KOA) patients. By investigating the effectiveness and feasibility of this approach, the trial holds the potential to enhance the accessibility and quality of care provided to individuals with KOA. The findings from this study could contribute valuable knowledge and insights to optimize the management of KOA and improve patient outcomes. Our trial incorporates the use of wearable devices to monitor exercise compliance, which is a notable strength. Adherence to exercise is a critical factor influencing clinical efficacy, particularly in the context of knee osteoarthritis (KOA) where patients lack direct supervision from doctors or physical therapists in home settings, unlike in outpatient settings. Thus, coaching and monitoring exercises become crucial elements when implementing telemedicine for managing KOA at home. However, there are certain limitations to consider. Firstly, it is not feasible to blind patients and physical therapists in our trial. Secondly, the trial requires the use of smartphones, which may pose a limitation in terms of accessibility for certain individuals. Lastly, while wearable devices provide valuable insights into exercise occurrence, they are unable to fully assess exercise accuracy. This limitation is inherent to the current technological capabilities and remains unresolved at present.

### Trial status

Recruitment will be started in July 2023, and it is estimated to have a total duration of 12 months. This protocol is version number 12 dated October 14, 2023.

### Supplementary Information 

**Additional file 1. **SPIRIT checklist

**Additional file 2. **Informed consent

## Abbreviations

KOA: Knee osteoarthritis
RCTs: Randomized controlled trials
TG: Trial group
CG: Control group
IMUs: Inertial Measurement Units
VAS: Visual analog scale
WOMAC: Western Ontario and McMaster Universities Osteoarthritis Index,
TUG: Time up go test
SF-36: MOS Item Short-Form Health Survey
PSEQ: Pain Self-Efficacy Questionnaire
DASS21: Depression-Anxiety-Stress Scale
TSK: Tampa Scale of Kinesiophobia
PCS: Pain Catastrophizing Scale
EARS: Exercise Adherence Rating Scale
SPIRIT: Standard Protocol Items: Recommendations for Interventional Trials
COA: Chinese Orthopedic Association
BMI: Body mass index
ITT: Intention-to-treat
PP: Per-protocol
SD: Standard deviation
MCID: Minimum clinically important difference

## Declarations

### Patient and public involvement

During the design phase, consultations were held with patients to gauge their confidence and expectations regarding the management of home-based rehabilitation facilitated by multi-component exercise therapy through telemedicine. A select group of patients was invited to provide feedback on the protocol, and the insights garnered from these consultations played a pivotal role in shaping the primary outcome and various other facets of the study. Moreover, an advisory committee was constituted right from the inception of project development. The responsibility for recruitment and execution of the study lies with the investigators stationed at each study site.

### Data access {29}

Access to the case report forms will be granted exclusively to the outcome assessors and the corresponding author. All entered data will be meticulously cross-verified by two independent assessors.

### Data sharing {31c}

There are no plans for granting public access to the full protocol, participant-level dataset, and statistical code.

### Biological specimens {33}

There are no plans for the collection, laboratory evaluation, and storage of biological specimens for genetic or molecular analysis in the current trial and for future use in ancillary studies.
