# Supplementary material for: Effect of the telemedicine-supported multicomponent exercise therapy in patients with knee osteoarthritis: study protocol for a randomized controlled trial
Source: Trials. 2023 Nov 14;24:729. doi: 10.1186/s13063-023-07749-4 (PMC10647045; doi:10.1186/s13063-023-07749-4)
Supplement: Supplementary file 2 — Additional file 2. Informed consent [file 13063_2023_7749_MOESM2_ESM.docx]

Patient Consent Form

Dear subject:

We invite you to participate in the "Clinical Study of Exercise Digital Therapy for Prevention and Treatment of Knee Osteoarthritis" approved by the mechanism of the optimal intervention parameters of pulsed electromagnetic fields promoting autophagy and repair of chondrocytes in knee osteoarthritis through regulation of oxidative stress and AMPK-SIRT1-PGC1α energy metabolism. The study will be carried out in Sichuan University West China Hospital and other hospitals, and an estimated 86 participants will voluntarily participate. This study has been reviewed and approved by the Biomedical Ethics Review Committee of West China Hospital, Sichuan University.

1. Why do we carry out this study?

Knee Osteoarthritis (Knee, KOA) is a major disabling disease worldwide, characterized by degeneration of the articular cartilage or subchondral bone damage that manifests as pain, swelling, and stiffness around the joint, and is the most common type of all arthritis. The clinical practice guidelines of the International OA organization emphasize the need to pay attention to the multiple aspects that cause pain and disability in KOA patients, and choose treatment options from multiple perspectives, including exercise therapy, patient education, weight loss, health guidance, self-management, and the change of perception of pain. It has been proven that integrating the various therapies recommended in the guidelines into a multidisciplinary combination therapy can bring more benefits to patients with KOA than physical therapy alone.

In China, most KOA patients are treated on an outpatient basis, but the duration of treatment is quite limited. In order to achieve good results, patients often need to exercise at home. However, structured exercise therapy and health guidance are inseparable from the guidance of professional rehabilitation personnel, and the combined use of various therapies is more inseparable from the support of professionals. Due to the lack of medical resources and patient compliance, it is difficult to carry out multi-disciplinary combination therapy in clinical application. Digital medical technology can provide technical support for doctors to deliver this combination therapy to patients, based on the Internet, the Internet of things, mobile devices, smartphones, websites and other devices can overcome the obstacles faced by traditional face-to-face treatment solutions, such as distance, time, economic factors, etc. Since the outbreak of COVID-19, patients with chronic musculoskeletal diseases, represented by KOA patients, have been severely affected in seeking outpatient treatment. The development of digital medicine can alleviate the shortage of medical resources, and can sustainably supervise the self-management of patients at home, promoting the long-term maintenance of the efficacy of treatment programs. However, only a small number of studies have explored the application of digital health to multidisciplinary combination therapy for KOA patients, and most studies have a high risk of bias, so the use of digital health application multidisciplinary combination therapy for KOA patients is still worthy of further exploration.

Therefore, we designed a single-blind randomized controlled trial to evaluate the efficacy of a multidisciplinary combination therapy based on digital health technology to improve pain and physical function in patients with knee osteoarthritis compared to a control group of patients who received online patient education and non-steroidal anti-inflammatory drugs. The aim is to provide a more convenient, low-consumption and effective method for clinical treatment of patients with knee osteoarthritis under the background of the new coronavirus epidemic and the era of big data, and promote self-home management of patients with knee osteoarthritis.

2. What do you need to do if you agree to participate in this study?

The study will last for 12 weeks and participants will be randomly assigned to one of two groups, to which you will be informed. The interventions in both groups were evidence-based treatments that did not involve invasive procedures.

(1) After you sign the informed consent, you need to truthfully answer the questions about your basic situation, including your age, height, weight, etc., as well as the general situation of knee osteoarthritis, we promise that your personal information and health conditions will be strictly confidential, only for the basis of judging your basic situation, and as the basis of scientific research reports;

(2) After the completion of basic data collection and baseline measurement, you will be randomly assigned to two groups to receive 12-week exercise digital therapy and daily care counseling respectively;

(3) During exercise training, you must ensure that other family members will supervise and care for you to ensure your safety during exercise training. If you are an elderly person living alone, we will arrange a nearby community medical institution to provide you with exercise supervision according to the situation;

(4) Exercise training three times a week, each time for 40-60 minutes, follow the exercise according to the APP's sports video guidance. For the first intervention, you need to evaluate, discuss and decide on a personalized exercise training program with a physical therapist offline, and receive personalized exercise guidance. For the first intervention, it is best to have your family members supervision or care;

(5) During the 12-week intervention period, you need to receive the illustrated patient education sent by the APP once a week. We will use professional checklists to provide you with educational information related to knee osteoarthritis according to the authoritative clinical guidelines;

(6) During the 12-week intervention period, you need to receive a weekly wechat video health guide interaction to help evaluate your weekly exercise progress and guide your lifestyle. The instructor will receive strict and professional training on knee osteoarthritis;

(7) During the 12-week intervention, you will need to receive 2 consecutive weeks of topical NSAID - Diclofenac diethylamine emulsion (GSK Consumer Healthcare S.A. Production, specifications 20g/ branch, product batch number DV6D, belongs to the approved drug), the drug use does not exceed the safe dose of the drug instructions, the drug amount according to the size of the affected area, 4 times/day, respectively along the front of the affected area, inside and outside the application sequence, gently rub, promote the absorption of the drug, the drug range should be greater than 10 mm at the distal end of the knee joint. Try to avoid contact with clothing within 10 minutes of medication, avoid bathing or exercise within 1 hour after medication, and choose the drug type and dosage according to the clinical practice guidelines of the American College of Rheumatology;

(8) The exercise regimen you use and your feelings during exercise training should be kept strictly confidential from other patients and clinical evaluators participating in the study, and you should also inform your family members to keep it strictly confidential;

(9) In week 0, Week 4, Week 8, and week 12, you need to be evaluated by the clinical evaluator through the APP, truthfully fill in the questionnaire or answer the evaluator's questions, each evaluation will take about 20-30 minutes, and your feelings and evaluation results during the evaluation should be kept confidential from the physical therapist and other participants in the study;

(10) During the 12-week intervention, other treatments outside the study intervention (such as non-study medication, physical therapy, etc.) are not affected, and you need to report to the clinical coordinator or physical therapist the timing, method, dosage, etc., of the treatment used;

(11) You will participate voluntarily throughout the study period, and if you withdraw for personal reasons, you will need to report and explain to the clinical Coordinator;

(12) Throughout the study period, if you have any condition lasting more than two days that requires other treatment, you should report it to your physiotherapist, clinical coordinator (tel: 18810218262) or program contact person (tel: 18991463887);

(13) For any health-related emergencies during the study period, you will need to report the incident, time and treatment/management to the Clinical Coordinator (tel: 1881 1021 18262) after reasonable assistance or management.

3. What are the treatment options available？

During the intervention period, no treatment other than the intervention was provided, and subjects may choose to use other treatments other than exercise therapy and daily care, but subjects should report their use of other treatments to the clinical research Assistant. If you can't find a dumbbell to use as a resistance training tool, you can also use a sandbag of the same weight tied to the far end of the limb to provide resistance.

4. These individuals are not suitable for participating in this trial:

If you meet any of the following criteria, you are not eligible to participate in this study:

(1) Knee arthroscopy or open surgery in the past 12 months, elective knee replacement.

(2) Combined with other joint problems (such as severe osteoporosis, rheumatoid arthritis, fractures, gout, joint tuberculosis, or joint tumors).

(3) The following diseases are confirmed or suspected: cerebral hemangioma, fatigue angina, severe anemia, installation of a fixed-frequency pacemaker, complete atrioventricular block.

(4) Uncontrolled hypertension or diabetes.

(5) Pregnant women.

(6) Patients with a history of mental disorders.

(7) Other unsupervised exercise is inappropriate (In addition to self-reported unsupervised participation, the Physical Activity Readiness Questionnaire (PAR-Q) will also be used to identify patients at risk for unsupervised participation in the trial).

(8) Supervision and care by other family members cannot be guaranteed for each exercise training.

(9) Cognitive impairment resulting in an inability to understand the physical therapist's instructions and the content of the online program (a score of < 24 on the Simple Intelligent Mental State Examination Scale MMSE is considered cognitive impairment).

5. What are the potential risks and possible adverse events of participating in the study?

5.1 The risks associated with the intervention involved in this clinical trial mainly include:

5.1.1 According to the whole process involved in exercise intervention, the directly relevant risks include:

(1) Insufficient warm-up before exercise leads to muscle strain and other sports injuries;

(2) During exercise, dizziness, chest pain, sudden cardiovascular and cerebrovascular diseases caused by unreasonable progress or weight, or falls and fractures due to irregular exercise methods or movements;

(3) There may be increased symptoms and muscle soreness after exercise;

5.1.2 Depending on where and how the exercise intervention is performed, the associated risks include:

(1) The main risks of exercise training in hospital under the supervision of physical therapists are the three aspects mentioned above;

(2) The subject conducts exercise training at home through a remote platform without face-to-face supervision by a physical therapist, and may also cause the above three risks due to non-standard movements;

(3) The mental health related risks caused by the decline in sports confidence and the deterioration of mood caused by the difficulty in meeting the expectations of the subjects or their inability to regularly adhere to sports training.

5.2 For possible risks, risk prevention and treatment mechanisms have been formulated:

5.2.1 Video link interactive health guidance course:

(1) Physiotherapists shall formulate personalized exercise plans for subjects through professional, detailed and personalized exercise tests, deliver correct and appropriate educational information, supervise subjects to perform adequate and reasonable warm-up activities, and implement exercise interventions according to subjects' exercise performance and health conditions. In addition, patients will be equipped with heart rate belts to monitor their exercise intensity. Prevent sports injuries and post-exercise symptoms directly related to the risk of exercise;

(2) When the subject has pain or aggravation of movement limitation symptoms and dizziness, etc., the APP will issue an alarm and forcibly exit the movement interface, and place the subject to rest until the symptoms are relieved. If the symptoms continue for more than 10 minutes and still cannot be relieved, According to the clinical manifestations according to the norms of relevant professional clinical rescue treatment immediately take corresponding treatment or rescue measures;

(3) The subject must exercise under the care or supervision of other family members, and in case of the above emergency, the family members will handle the emergency or make an emergency call;

(4) Subjects who are not supervised by other family members during the exercise training related to the project will be arranged to the nearest community medical institution for exercise training, and corresponding treatment or rescue measures will be taken immediately in accordance with the relevant professional clinical rescue treatment norms when falls, chest pain, cardiovascular and cerebrovascular disease emergencies occur in the hospital environment;

(5) Emergency contact number: 112 (first aid), 18810218262 (clinical coordinator), 18991463887 project contact person).

5.2.2 Subjects' home exercise training:

(1) The physiotherapist will inform the subject to conduct exercise training including warm-up, training, cooling, stretching and other steps in strict accordance with the personalized exercise program, and carry out reasonable exercise progression according to their activity tolerance, exercise performance and irritability of symptoms, and each progression should be carried out after reaching an agreement with the physiotherapist. To prevent sports injuries caused by improper exercise and other directly related risks;

(2) The subject will conduct exercise training under the care or supervision of other family members, and in case of the above emergency, the family members will handle the emergency or make an emergency call;

(3) The subjects need to record the adverse events and pain degree through the exercise diary of the remote platform. The clinical coordinator will check the self-assessment results of the patients participating in the remote exercise training at a fixed time every day. When the pain is found to be severely aggravated or the record is missing, the coordinator will call for inquiry;

(4) Emergency contact number: 112 (first aid), 18810218262 (clinical coordinator), 18991463887 project leader).

**Please note:**

1. You must ensure the safety and stability of the exercise training environment, regardless of whether you have video connection with a physiotherapist, and you must conduct exercise training under the supervision and care of family members to ensure safety;

2. If you are an elderly person living alone, we will arrange for you to go to a nearby community medical institution for exercise training under the supervision of professionals.

3.You must strictly follow the physiotherapist's personalized exercise plan, which is customized according to your condition, evaluation results and exercise performance, and any further steps and changes to the exercise plan need to be discussed with the physiotherapist to prevent exercise-related injuries;

4. When you have a sports injury while exercising at home, you need to perform a series of emergency treatment methods such as cold compress braking, elevation and pressure dressing for sports injury. Specific methods will be demonstrated and taught before the intervention begins;

5．When you appear in the process of exercise symptoms worsen, dizziness and other symptoms, should stop training, rest 10 minutes after the symptoms still do not improve should immediately enter the medical emergency procedure, you can call the emergency telephone, emergency contact number above;

When the above risks occur during treatment, adverse events caused by this study will be handled by the researcher free of charge. For serious adverse events that are judged to be related to clinical trials, the researcher will treat them free of charge. The research team will provide economic compensation of corresponding value for the economic losses caused, including transportation subsidies and accommodation subsidies of 300 yuan. Proof of loss with legal value shall prevail.

6. What are the benefits you can take from the study?

By participating in this study, you may be able to improve your condition, mainly in terms of improved function, pain relief, scientific self-management habits, and improved mental health and quality of life. This research could also help determine which treatments are safer and more effective in treating other patients with conditions similar to yours. But these benefits are not guaranteed.

7. Are there any fees that need to pay during the study?

Participants will not be required to pay any fees to participate in the study. Participants who complete all interventions and evaluations during the intervention period will be given a transportation allowance of 300 yuan and a room and board allowance.

8. Is personal information confidential?

Your research data will be stored at West China Hospital of Sichuan University, and your medical records will be accessible to investigators, study authorities, and ethics review committees. Any public reporting of the results of this research will not disclose your personal identity. We will make every effort to protect the privacy and personal information of your personal medical data to the extent permitted by law.

9. Must I participate in a study?

Participation in the study is completely voluntary and you may refuse to participate in the study or withdraw from the study at any stage of the study without discrimination or retaliation, without affecting your medical treatment and rights. If you decide to withdraw from the study, please contact your physical therapist or program manager for proper diagnosis and treatment. Project leader Tel: 18991463887 (Yuan-Feng).

Patient Statement:

I have read the above presentation on this study, and my researchers have fully explained to me the purpose of this study, its operational process, and the possible risks and potential benefits of participating in this study and answered all my relevant questions.

I understand the purpose of this study and I am free to withdraw at any time without medical cares or legal rights being affected. I am voluntary to participate in this study. I understand that results of my visits may be shared with the research team of West China Hospital of Sichuan University.

I agree to allow any information provided to be medical research upon the understanding that my identity will remain anonymous wherever possible.

Please indicate your wishes in the below scenarios:

Please tick or initial yes or no: Please tick √ or initial

YES NO

I agree for my details to be shared and used in further

Research that be running by West China Hospital of Sichuan

University.

Patient (to be completed by the patient):

Signature: ___________________________________________________________

Name (block letters): ___________________________________________________

Date: _______________________________________________________________

Phone: ______________________________________________________________

Legal representative (block letters, if applicable): _____________________________

Relationship with patient: ________________________________________________

Witness (block letters, if applicable): _______________________________________

Date: _______________________________________________________________

Investigator Statement:

I have explained the request to the above-named patient, particularly, the ethical principles, risks, benefits, free, voluntariness and confidentiality that may arise from participating in this study. And he/she has indicated his/her willingness for participating in this study.

Signature: ___________________________________________________________

Name (block letters): ___________________________________________________

Date: ________________________________________________________________

Ethics Committee on Biomedical Research,

West China Hospital of Sichuan University

Tel: 028-85422654!028-85423237

(1 copy for patient; 1 held in patient notes, original stored in Investigator Site File)
